# Peptidomic data of egg white gastrointestinal digests prepared using the Infogest Harmonized Protocol

**DOI:** 10.1016/j.dib.2020.105932

**Published:** 2020-06-26

**Authors:** Marta Santos-Hernández, Beatriz Miralles, Lourdes Amigo, Isidra Recio

**Affiliations:** Instituto de Investigación en Ciencias de la Alimentación, CIAL (CSIC-UAM, CEI UAM+CSIC), Nicolás Cabrera, 9, 28049 Madrid, Spain

**Keywords:** Egg white protein, Characterization, Mass spectrometry, Maldi-tof, Peptidomics

## Abstract

These data are related to the research article entitled “Induction of CCK and GLP-1 release in enteroendocrine cells by egg white peptides generated during gastrointestinal digestion”. In this article, the peptide and free amino acid profile of egg white gastrointestinal *in vitro* digests is shown. Egg white proteins were digested following the INFOGEST gastrointestinal digestion protocol. Different time points of gastric and intestinal digestion were characterized regarding protein, peptide and amino acid content. Protein degradation was followed by SDS-PAGE where some electrophoretic bands were identified by MALDI-TOF/TOF after tryptic digestion. Moreover, the molecular weight distribution of egg white peptides found at different times of gastrointestinal digestion was performed using MALDI-TOF. Peptides identified from the most abundant egg white proteins by tandem mass spectrometry were represented using a peptide profile tool and raw data are given in table format. These results reveal the protein regions resistant to digestion and illustrate the free amino acid profile of egg white protein at the end of the digestion process. These data can be used for nutritional purposes and to identify allergen epitopes or bioactive sequences.

**Specifications Table**

**Table:** 

Subject area	Biochemistry
More specific subject area	Proteomics and biochemistry
Type of data	Figures, Tables
How data was acquired	High-pressure liquid chromatography coupled to electron spray ionization interface and ion trap. Matrix assisted Laser Desorption/Ionization coupled to a time of flight detector
Data format	Raw Analyzed
Parameters for data collection	Digested samples following INFOGEST protocol were freeze-dried and kept at −20 °C until analysis. Digests were analyzed by mass spectrometry after a reducing step with dithiothreitol.
Description of data collection	MS/MS raw files were processed by using Data Analysis (version 4.0 Bruker Daltonics) and Biotools version 3.2, and the identification search was achieved using Mascot v2.4
Data source location	Data is collected and analysed at the Institute of Food Science Research, CIAL (CSIC-UAM). Nicolás Cabrera 9, 28049, Madrid, Spain
Data accessibility Related research article	Data in this article M. Santos-Hernández, L. Amigo, Recio, I. “Induction of CCK and GLP-1 release in enteroendocrine cells by egg white peptides generated during gastrointestinal digestion”.

Value of the data

• The data provide the distribution of the nitrogen fraction into peptides and amino acids at the end of the gastrointestinal digestion of egg white. The profile of free amino acids that can be used for nutritional purposes is also given.
• The here provided proteomic, peptidomic and amino acid profiles of egg white protein digests can be compared with *in vivo* data or with data obtained in dynamic systems.
• Egg white protein domains resistant to gastrointestinal digestion are provided which could serve to detect allergen epitopes or peptides with biological activities.
• This peptidomic characterization was useful to identify peptides as inducers of incretin hormones, being relevant to control food intake and diabetes.

## 1 Data description

Egg white protein was digested following a harmonized *in vitro* digestion protocol [1,2] where samples were taken at 30 and 120 min of gastric digestion and 30 and 120 min of gastrointestinal digestion. These digests were centrifuged at 5000 × *g* over 20 min to separate soluble and insoluble fraction, followed by snap freezing in liquid nitrogen and freeze-dried. All digests were characterized regarding their protein, peptide and amino acid composition.

**Fig. 1:**
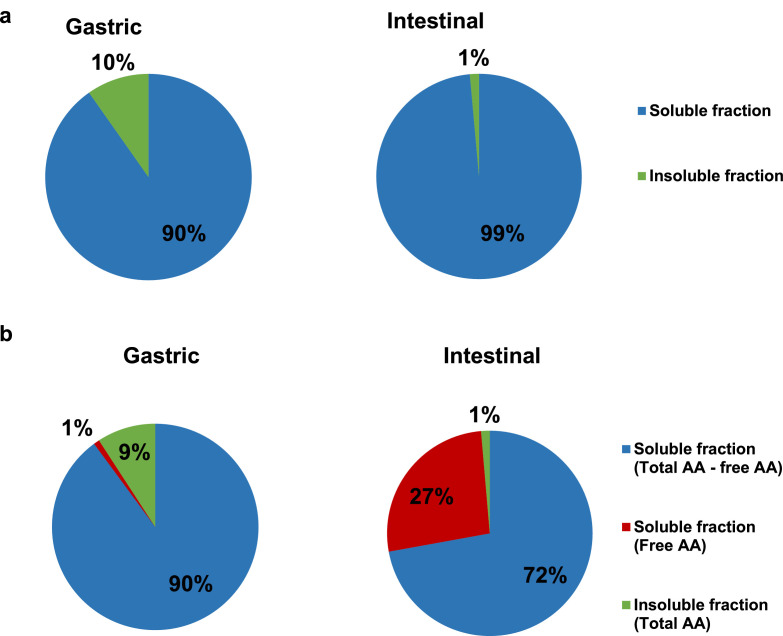
Distribution of the nitrogen content between soluble and insoluble fraction after gastric and intestinal digestion. Digests were centrifuged at 5000 × *g* for 20 min. Supernatant and pellet were freeze-dried and weighted. Nitrogen content in each fraction was determined by a) elemental analysis and b) amino acid analysis. Total and free amino acids were separately determined in the soluble fraction.

The distribution of the nitrogen fraction after egg white *in vitro* gastric and intestinal digestion was assessed by elemental analysis (Fig. 1a) and amino acid analysis (Fig. 1b). Around 90% of the total nitrogen content was collected in the soluble fraction after gastric digestion, determined by elemental analysis, and this percentage increased up to 99% at the end of the intestinal phase (Fig. 1a). Similar percentages were obtained by total amino acid analysis where the soluble fraction represented 91% and 99% at the end of the gastric and the intestinal phase, respectively (Fig. 1b). At the end of the gastric phase, just 1% of the total nitrogen fraction was in the form of free amino acids, increasing up to 27% at the end of the intestinal phase. The difference between total and free amino acids in the soluble part corresponded to the nitrogen fraction in the form of proteins and peptides which ranged from 90% at the end of the gastric phase to 72% at the end of the intestinal phase.

**Fig. 2:**
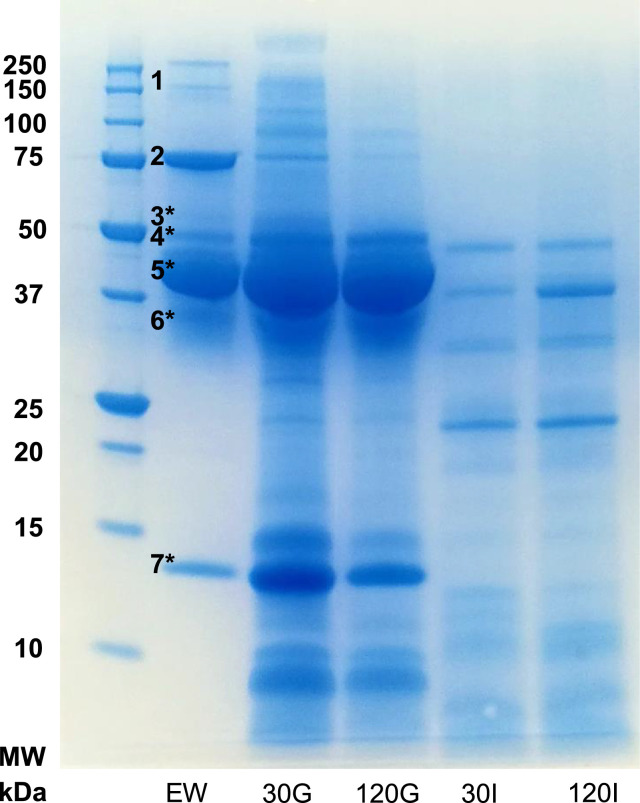
SDS-PAGE protein profiles of egg white protein at different times of *in vitro* gastrointestinal digestion. MW, molecular weight marker; EW, egg white undigested protein; 30 G and 120 G, 30 and 120 min gastric digestion; 30I and 120I, 30 and 120 min intestinal digestion. The numbers correspond to an identified band. 1, Ovomucin; 2, Ovotransferrin; 3, Ovoinhibitor; 4, Ovalbumin-Y; 5, Ovalbumin; 6, Ovomucoid; 7, Lysozyme. The electrophoretic bands with * were identified by MALDI-TOF/TOF after hydrolysis with trypsin.

Protein degradation during egg white gastrointestinal digestion was followed by SDS-PAGE (Fig. 2). At the end of the gastric phase, main electrophoretic bands were identified as ovalbumin and ovalbumin-related protein Y and these were still detected at the end of the intestinal phase. Electrophoretic bands with * in Fig. 2 were identified by MALDI-TOF/TOF after in-gel digestion with trypsin.

**Fig. 3:**
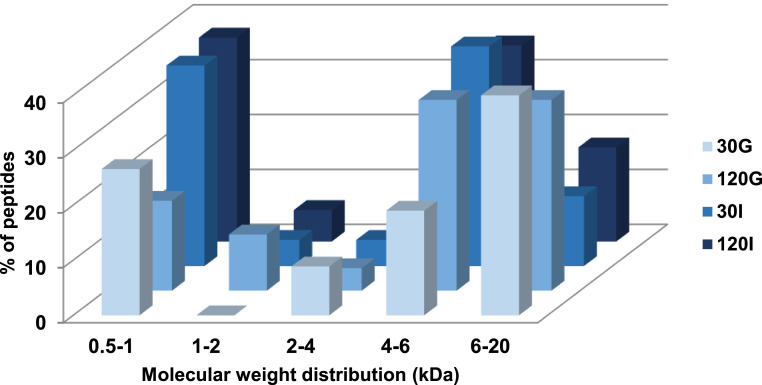
Molecular weight distribution of egg white peptides found at different times of gastrointestinal digestion. 30 G and 120 G, 30 and 120 min of gastric digestion, respectively; 30I and 120I, 30 and 120 min intestinal digestion, respectively.

**Fig. 4:**
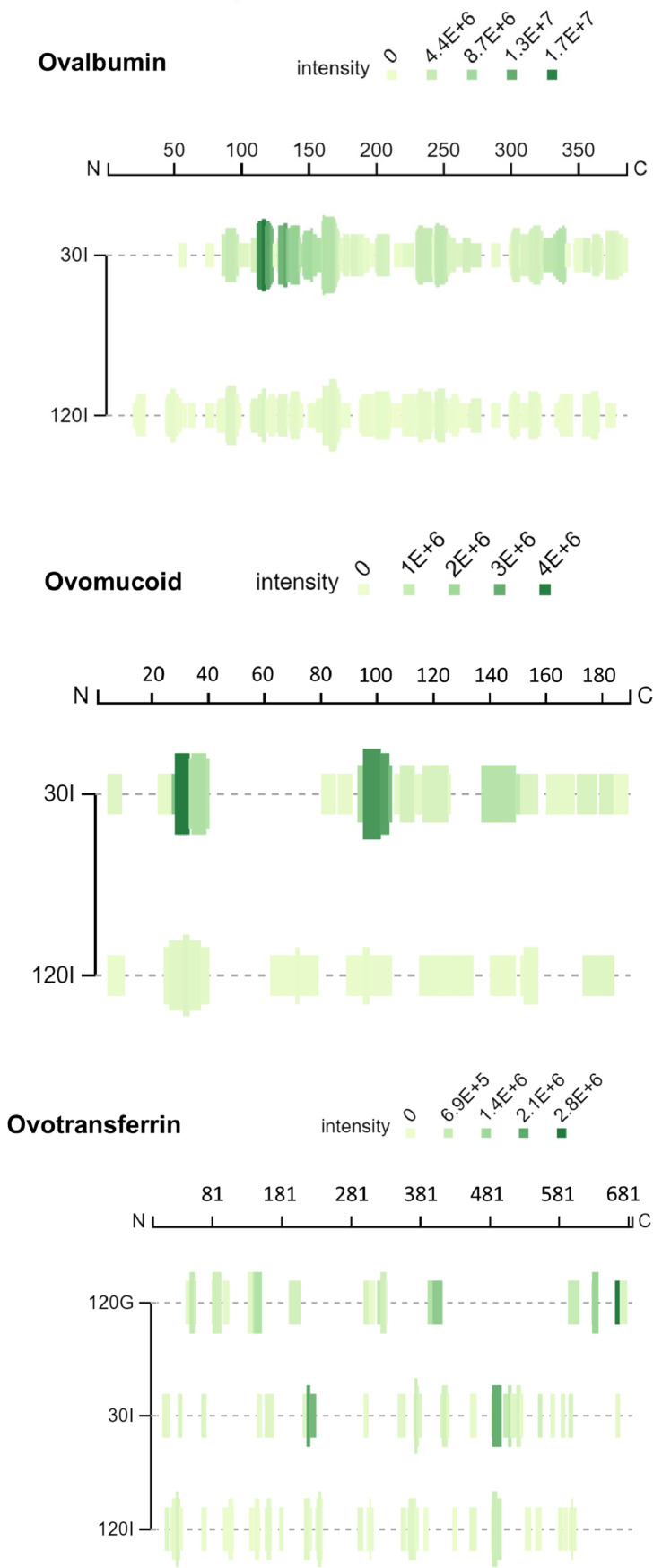
Peptides from ovalbumin, ovomucoid and ovotransferrin identified in egg white gastrointestinal digests represented using the Peptigram Bioware tool. Each vertical bar corresponds to an amino acid identified as part of a peptide sequence. The height of the bar is proportional to the number of the peptides overlapping this position and the color intensity is proportional to the sum of the intensities of the peptides overlapping a given position. Each line corresponds to a different time point: 120 G, 120 min gastric digestion; 30I and 120I, 30 and 120 min intestinal digestion, respectively.

**Fig. 5:**
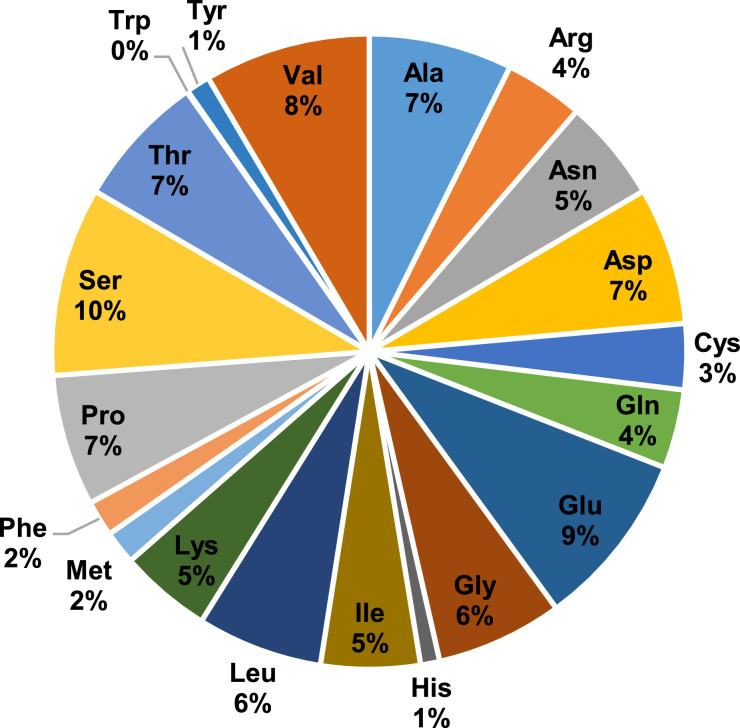
Amino acid composition of the identified peptide sequences at the end of the *in vitro* gastrointestinal digestion of egg white. Repeated sequences are avoided.

Egg white protein digests were also characterized by MALDI-TOF and the peptide profile is represented in Fig. 3, which describes the percentage of peptides within a given molecular weight range. As expected, peptides with a molecular weight lower than 2 kDa increased during gastrointestinal digestion and the amount of longer peptides decreased. However, it should be noted that peptides with a molecular weight higher than 6 kDa were still detected at the end of gastrointestinal digest. Peptidomic characterization of the digests was performed by HPLC tandem mass spectrometry (HPLC-MS/MS). Raw data of peptide sequences identified at the end of the gastrointestinal digestion by HPLC-MS/MS are given in Tables 1 to 5. The identified peptides from the major protein fractions, ovalbumin, ovomucoid and ovotransferrin, at different time points during gastrointestinal digestion, are also represented using the peptide profile tool from the Peptigram web-based application (Fig. 4). In these graphs, each vertical bar corresponds to an amino acid identified as part of a peptide sequence, the height of the bar is proportional to the number of peptides overlapping this position and the color intensity is proportional to the sum of the intensities of the peptides overlapping a given position. Under our mass spectrometry conditions, peptides with a molecular weight between 5 and 30 kDa are detected. The blank regions observed in the peptide profile during the gastric phase probably correspond to peptide fragments too long to be solubilized or ionized under our analysis conditions, while blank regions in the intestinal phase are more likely due to short peptides, free amino acids, or peptides with low ionization capacity. It has to be noted that peptide intensity depends on peptide ionization capacity and abundancy, and in consequence, peptide intensity cannot directly be transformed to peptide concentration. During the gastric phase, only peptides from ovotransferrin were identified, suggesting a higher susceptibility of this protein to the action of pepsin. Several ovalbumin and ovomucoid peptides were detected after 30 and 120 min intestinal digestion. In addition, the amino acid composition of the identified peptides was analysed by using the ExPASy-Protparam tool (Fig. 5). Resistant peptides identified at the end of intestinal digestion by mass spectrometry were rich in serine, valine and in negatively charges residues aspartic and glutamic acid.

**Table 1:** Ovalbumin-derived peptides identified at the end of egg white gastrointestinal digestion.

RANGE^a^		SEQUENCE^b^	RANGE^a^		SEQUENCE^b^
20	28	KVHHANENI	188	199	AFKDEDTQAMPF
22	28	HHANENI	190	198	KDEDTQAMP
23	28	HANENI	200	209	RVTEQESKPV
44	50	LGAKDST	200	211	RVTEQESKPVQM
44	52	LGAKDSTRT	200	204	RVTEQ
45	50	GAKDST	200	210	RVTEQESKPVQ
48	52	DSTRT	205	209	ESKPV
48	55	DSTRTQIN	213	217	YQIGL
54	58	INKVV	219	228	RVASMASEKM
61	65	DKLPG	220	229	VASMASEKMK
74	79	CGTSVN	220	228	VASMASEKM
82	97	SSLRDILNQITKPNDV	230	234	ILELP
83	90	SLRDILNQ	230	240	ILELPFASGTM
89	97	NQITKPNDV	230	242	ILELPFASGTMSM
89	95	NQITKPN	230	241	ILELPFASGTMS
89	99	NQITKPNDVYS	232	240	ELPFASGTM
90	95	QITKPN	232	241	ELPFASGTMS
91	95	ITKPN	243	250	LVLLPDEV
107	117	YAEERYPILPE	244	252	VLLPDEVSG
108	117	AEERYPILPE	244	250	VLLPDEV
113	117	PILPE	244	253	VLLPDEVSGL
116	124	PEYLQCVKE	246	250	LPDEV
116	125	PEYLQCVKEL	254	260	EQLESII
121	125	CVKEL	255	260	QLESII
127	134	RGGLEPIN	257	261	ESIIN
127	133	RGGLEPI	261	267	NFEKLTE
128	133	GGLEPI	269	277	TSSNVMEER
136	142	QTAADQA	271	277	SNVMEER
136	143	QTAADQAR	286	291	MKMEEK
137	142	TAADQA	299	303	MAMGI
137	145	TAADQAREL	299	306	MAMGITDV
150	161	VESQTNGIIRNV	300	306	AMGITDV
156	160	GIIRN	302	306	GITDV
161	165	VLQPS	308	321	SSSANLSGISSAES
161	172	VLQPSSVDSQTA	314	319	SGISSA
161	170	VLQPSSVDSQ	314	322	SGISSAESL
161	169	VLQPSSVDS	314	321	SGISSAES
161	173	VLQPSSVDSQTAM	333	345	AEINEAGREVVGS
163	170	QPSSVDSQ	336	345	NEAGREVVGS
166	170	SVDSQ	337	345	EAGREVVGS
166	174	SVDSQTAMV	354	358	SVSEE
166	173	SVDSQTAM	354	365	SVSEEFRADHPF
166	172	SVDSQTA	354	364	SVSEEFRADHP
176	180	VNAIV	360	364	RADHP
188	198	AFKDEDTQAMP	360	365	RADHPF

a Range in the protein. Uniprot accession number: P01012.

b One letter amino acid code is used.

**Table 2:** Ovomucoid-derived peptides identified at the end of egg white gastrointestinal digestion.

RANGE^a^		SEQUENCE^b^
1	6	AEVDCS
21	36	VCNKDLRPICGTDGVT
21	29	VCNKDLRPI
23	33	NKDLRPICGTD
28	36	PICGTDGVT
59	68	DGECKETVPM
68	75	MNCSSYAN
86	93	LCNRAFNP
92	101	NPVCGTDGVT
137	145	DCSEYPKPD
148	153	AEDRPL
149	153	EDRPL
170	180	AVVESNGTLTL

a Range in the mature form of the protein. Uniprot accession number: P01005.

b One letter amino acid code is used.

**Table 3:** Ovotransferrin-derived peptides identified at the end of egg white gastrointestinal digestion.

RANGE^a^		SEQUENCE^b^	RANGE^a^		SEQUENCE^b^
13	17	SSPEE	304	308	AIMLK
21	30	NNLRDLTQQE	308	312	KRVPS
24	37	RDLTQQERISLTCV	353	359	DEKSKCD
29	33	QERIS	363	373	VVSNGDVECTV
66	71	EAGLAP	365	372	SNGDVECT
97	110	VVKKGTEFTVNDLQ	373	377	VVDET
105	109	TVNDL	386	391	KGEADA
135	139	RGAIE	428	432	PASYF
142	147	GIESGS	452	460	KSCHTAVGR
143	148	IESGSV	483	490	YFSEGCAP
157	165	SASCVPGAT	484	496	FSEGCAPGSPPNS
160	164	CVPGA	485	496	SEGCAPGSPPNS
178	182	PKTKC	532	539	VEKGDVAF
214	220	NENAPDQ	547	554	ENTGGKNK
214	223	NENAPDQKDE	580	584	DYREC
230	238	DGSRQPVDN	587	591	AEVPT
232	236	SRQPV	595	599	VVRPE
234	238	QPVDN	599	604	EKANKI
291	297	KDPVLKD			

a Range in the mature form of the protein. Uniprot accession number: P02789.

b One letter amino acid code is used.

**Table 4:** Lysozyme-derived peptides identified at the end of egg white gastrointestinal digestion.

RANGE^a^		SEQUENCE^b^
18	25	DNYRGYSL
34	44	FESNFNTQATN
39	43	NTQAT
44	52	NRNTDGSTD
45	52	RNTDGSTD
47	52	TDGSTD
64	72	CNDGRTPGS
72	83	SRNLCNIPCSAL
85	90	SSDITA
85	89	SSDIT
97	105	KIVSDGNGM
99	105	VSDGNGM
117	122	GTDVQA

a Range in the mature form of the protein. Uniprot accession number: P00698.

b One letter amino acid code is used.

**Table 5:** Mucin-5B-derived peptides identified at the end of egg white gastrointestinal digestion.

RANGE^a^		SEQUENCE^b^	RANGE^a^		SEQUENCE^b^
34	38	GRSEC	1064	1073	EACHSKVNPI
98	102	VILEV	1165	1169	GCYPE
125	129	IEDTC	1216	1223	YPLNETIY
125	131	IEDTCAY	1235	1241	FCGPNGM
134	141	VTSKLGLT	1326	1330	EALET
147	154	ADTLLLDL	1384	1388	CLGEE
200	204	EKCPD	1528	1532	GIRIT
272	277	CICSTL	1580	1584	KSDDA
293	298	EWRTKE	1581	1590	SDDARKRNGE
376	380	STPCQ	1596	1600	KEMAL
433	437	TFVVI	1648	1660	PPQPYYEACVASR
453	457	KNVLV	1690	1694	RGQTN
454	458	NVLVT	1721	1729	REVIVDTLL
545	552	FRTATGAV	1744	1751	PDGNILLN
546	551	RTATGA	1827	1834	TETVCECD
550	562	GAVEDSAAAFGNS	1827	1834	TETVCECD
657	662	QGICDP	1880	1884	KPGAV
753	761	DCIGETVLV	1880	1887	KPGAVVPK
901	908	DAGTFRIV	1886	1896	PKSSCEDCVCT
923	928	LKITLI	1910	1914	CVPVK
1012	1019	GQSVEMSI	1913	1917	VKCQT
1044	1051	QPFKSALG			

a Range in the mature form of the protein Uniprot accession number: Q98UI9.

b One letter amino acid code is used.

**Fig. 6:**
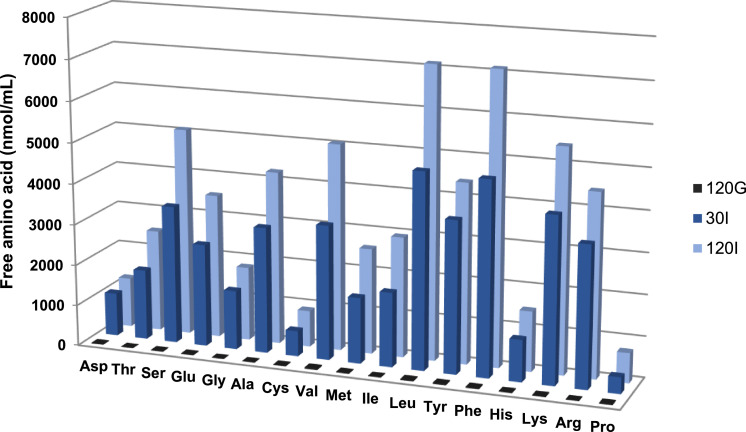
Free amino acid profile in egg white protein digests at different time points. 120 G corresponds to 120 min of gastric digestion; 30I and 120I correspond to 30 and 120 min of intestinal digestion, respectively.

Free amino acids released after 120 min of gastric digestion and 30 and 120 min of intestinal digestion were followed by HPLC and post-column ninhydrin derivatization. As shown in Fig. 6, free amino acids were mainly released during intestinal digestion, with phenylalanine, leucine and lysine being the most abundant, followed by arginine, valine and serine.

## 2 Experimental design, materials, and methods

### 2.1 Distribution of nitrogen content

Supernatant and pellet from gastric and intestinal digests were freeze-dried and weighted. Protein content in each fraction was measured by elemental analysis and by total amino acid analysis after acid hydrolysis with HCl 6 N at 110 °C for 24 h. In addition, free amino acids were also determined in the soluble part according to the method previously published [3]. The difference between total and free amino acids was ascribed to proteins and peptides.

### 2.2 Molecular weight distribution by MALDI-TOF

Samples were diluted with 33% acetonitrile and 0.1% of trifluoroacetic acid prior to spotted into a MALDI target plate with 2,5-dihydroxybenzoic acid matrix. Analyses were performed on an Autoflex SpeedTM (Bruker Daltonic, Bremen, Germany). Mass spectra were acquired in positive reflection mode and were collected from the sum of 1000 on average lasers shots. Monoisotopic peaks were generated by FlexAnalysis software. The monoisotopic peaks were organized and represented in a molecular weight distribution range. All other methods are described in [3].

## Declaration of Competing Interest

The authors declare that they have no known competing financial interests or personal relation-ships that could have appeared to influence the work reported in this paper.
